# *Lactobacillus crispatus*-derived nCEV vesicles promote cutaneous wound healing and inhibit HPV16 infection

**DOI:** 10.1128/msystems.00683-25

**Published:** 2025-08-19

**Authors:** Ke Peng, Xiaoting Chen, Yulin Zhang, Lina Liu, Qingye Huang, Shujuan Du, Caixia Zhu, Yuyan Wang, Fang Wei, Shujun Gao, Qiliang Cai

**Affiliations:** 1 Center of diagnosis and treatment for cervical & uterine cavity disease, Obstetrics and Gynecology Hospital of Fudan University, Shanghai Key Laboratory of Female Reproductive Endocrine-Related Disease, & MOE&NHC&CAMS Key Laboratory of Medical Molecular Virology, Shanghai Institute of Infectious Disease and Biosecurity, Shanghai Frontiers Science Center of Pathogenic Microorganisms and Infection, School of Basic Medical Science, Qidong-Fudan Innovative Institute of Medical Science, Shanghai Medical College, Fudan University 12478, Shanghai, China; 2 ShengYushou Center of Cell Biology and Immunology, School of Life Sciences and Biotechnology, Shanghai Jiao Tong University 12474, Shanghai, China; 3 Department of Gynecology, the International Peace and Child Health Hospital, School of Medicine, Shanghai Jiao Tong University 12474, Shanghai, China; 4 Expert Workstation, Baoji Central Hospital, Baoji, China; Universiteit Leiden, Leiden, the Netherlands

**Keywords:** *Lactobacillus*, nCEVs, D-lactate, wound-healing, HPV infection

## Abstract

Cervical cancer (CC) is a common gynecological malignancy, primarily associated with persistent infection by high-risk human papillomavirus (HPV). The vaginal microbiota, dominated by *Lactobacillus* species, plays a critical role in maintaining a healthy vaginocervical microenvironment. However, the influence of *Lactobacillus* on the pathogenesis and treatment of CC remains underexplored. Here, we report that the optimized 60–80 nm size dominant of extracellular vesicles from various *Lactobacillus* species (nLEVs) significantly affects cutaneous wound healing and HPV infection. Specifically, nCEVs *from Lactobacillus crispatus* demonstrate superior efficacy compared to nIEVs from *Lactobacillus iners* in promoting cell migration, angiogenesis, and wound healing via macrophage M2 polarization and blocking intravaginal HPV16 infection. Moreover, metabolic profiling revealed that D-lactate may be key to the functions of nCEVs. Altogether, our findings uncover a novel nCEV/D-lactate-mediated mechanism that promotes homeostasis and offers potential new approaches for the prevention and treatment of CC.

**IMPORTANCE:**

This study identifies *Lactobacillus crispatus*-derived vesicles (nCEVs) as crucial for cervicovaginal homeostasis, functioning through promoting wound healing via macrophage polarization and blocking HPV infection, and delivering D-lactate—a key bioactive component. These insights advance microbiome-based female healthcare interventions.

## INTRODUCTION

Cervical cancer (CC) ranks as the fourth-most common malignant tumor among women (1). Persistent infection with high-risk human papillomavirus (HR-HPV) is the primary cause of CC (2). Annually, over 600,000 new cases of CC are diagnosed worldwide, resulting in approximately 300,000 deaths (1, 3). Prophylactic vaccines have demonstrated efficacy in preventing many HR-HPV infections; they do not cover all high-risk types, and global vaccination rates remain disappointingly low. In response to this major public health challenge, an international initiative has been launched to eliminate CC through an ambitious series of public health initiatives (4). Despite these efforts, effective therapeutic strategies, particularly interventions against persistent high-risk HPV infections and CC, are limited so far and remain to be further investigated.

The human female vagina acts as a permeable canal, shielded from the external environment by the vaginal epithelial barrier (5). This barrier includes four primary protective measures: mechanical, immune, microbial, and chemical barriers. Among these, the mechanical barrier, often referred to as the physical barrier, consists of a continuous layer of vaginal epithelial cells that are linked by tight junctions and desmosomal proteins (6, 7). The microbial barrier is primarily sustained by over 50 types of microorganisms (8, 9), with lactobacilli accounting for 70%–80% of the vaginal microbiome. The Ravel group identified five distinct community state types (CSTs) within the vaginal microbiome through high-throughput 16S rRNA sequencing (10). Four of these CSTs are predominantly composed of *Lactobacillus* species: *Lactobacillus crispatus* (*L. crispatus,* regarded as the healthiest), *Lactobacillus iners* (*L. iners)*, *Lactobacillus gasseri* (*L. gasseri*), and *Lactobacillus jensenii* (*L. jensenii)* (10, 11). The colonization of the vaginal mucosa by *Lactobacillus* is critical in reducing the risk of urogenital infections as it helps prevent the adhesion, attachment, and invasion of pathogens (12, 13). The fifth CST is characterized by a significant reduction in *Lactobacillus* and an increase in a heterogeneous group of anaerobes, including *Gardnerella vaginalis* (*G. vaginalis*) (10, 14). This state, often referred to as a non-*Lactobacillus*-dominated state or a state of high diversity, is closely associated with clinical bacterial vaginosis (BV) (15). Among these species, *L. iners* stands out as possessing the smallest genome of all known lactobacilli; it exhibits some differences compared to other major vaginal *Lactobacillus* species. The predominance of *L. iners* is associated with transitioning from *Lactobacillus* dominance to the development of diverse bacterial communities linked to BV (16). Furthermore, *L. iners* has been found to encode a translational inhibitor, which is speculated to assist in its competition with other vaginal *Lactobacillus* (17). Vaginal dysbiosis is frequently associated with increased mucosal inflammation, activation of immune cells, and reduced mucosal wound-healing capacity (18). Research has revealed an association between *G. vaginalis* and *L. iners* with pro-inflammatory conditions (19), whereas *L. crispatus*, *L. jensenii*, and *L. gasseri* are more commonly associated with non-inflammatory states and reduced levels of pro-inflammatory cytokines (20). The vaginal epithelial barrier and the immune condition are vitally important in HPV infection and clearance (21). Increasing evidence points to a meaningful relationship between vaginal microbiomes and HPV infection (22). Recent meta-analyses indicate that women with *Lactobacillus*-enriched microbiomes are less likely to acquire HPV infection compared to those with microbiomes associated with BV (22–24). In addition, research suggests that women with a low relative abundance of vaginal *Lactobacillus* species and a high proportion of BV-associated bacteria face greater challenges in clearing HPV infections. Specifically, a higher abundance of *L. crispatus* has been linked to successful HPV clearance (25), whereas an *L. iners*-dominant microbiome is often found in women with cervical intraepithelial neoplasia (CIN) (22, 23). Nevertheless, the potential role of these microbiomes in the recurrence and persistence of HPV infections remains unclear.

It has been shown that both commensal gram-negative and gram-positive bacteria can secrete bacterial extracellular vesicles (bEVs) and engage with host cells (26). While it was believed for many years that gram-positive bacteria could not release EVs due to their thick cell walls (26). However, Lee et al. reported that *Staphylococcus aureus* can also release EVs (27), and EVs have been successfully isolated from several strains of *Lactobacillus* (26, 28). These EVs play a vital role in numerous physiological and pathological processes as they transport bioactive molecules, including proteins, DNA, and RNA, which can alter the biological properties of both bacteria and host cells (29). Although the role of gram-positive bEVs has been less extensively studied compared to mammalian or gram-negative EVs (28, 29), increasing evidence has indicated that *Lactobacillus*-derived EVs (LEVs) may provide beneficial effects for the host (28, 30). Particularly, LEVs have been linked to controlling pathogen infectivity, regulating host immunity, maintaining epithelial integrity, and inducing apoptosis in hepatic cancer cells (12, 28, 29, 31). Despite their importance, only a few studies have focused on LEVs in the vaginal niches (12, 32–35), and the mechanisms of their biogenesis, composition, and functions remain to be further investigated.

Although antibiotics and antiviral medications are widely utilized in the effective treatment of sexually transmitted diseases (STDs) (36, 37), the success rate for BV treatment is only approximately 50%, and the recurrence rate exceeds 50% within 6 to 12 months (38). Accumulating evidence shows that factors contributing to these outcomes include drug resistance, challenges in eliminating polymicrobial biofilms, difficulties in restoring an acidic pH level, and obstacles in re-establishing *Lactobacillus* dominance. It is vital to seek effective and safe treatments that can restore vaginal microecology and repair the vaginal barrier. Emerging research underscores the relationship between LEVs and health benefits associated with probiotic bacteria, highlighting that LEVs could be a key mediator of communication across different biological kingdoms (39–42). In this study, we optimized the production of LEVs with 60–80 nm size dominance (nLEVs) under different conditions, which were derived from *L. crispatus* (nCEVs), *L. jensenii* (nJEVs), or *L. iners* (nIEVs). We found that nCEVs, instead of nJEVs or nIEVs, efficiently promote cell migration, junction integrity, and angiogenesis *in vitro*, particularly induction of wound healing, activation of M2 macrophages, and inhibition of HPV16 intravaginal infection in a mouse model. Moreover, we revealed that the D-lactate, presented in nCEVs not IEV, enhances wound healing and blocks viral infection for cutaneous homeostasis. These findings provide new avenues for applying probiotic LEVs in regenerative medicine, particularly in the therapy of cervical cancers.

## MATERIALS AND METHODS

### *Lactobacillus* strains

All *Lactobacillus* strains of vaginal origin, *L. crispatus* strain cgmcc1.2743 (SHBCC D51652), *L. jensenii* strain 62G (ATCC 25258), and *L. iners* strain AB107 (ATCC 55195), were cultured at 37°C in MRS (de Man, Rogosa, and Sharpe) broth (Solarbio), supplemented with 0.05% L-cysteine (Sangon Biotech). We achieved anaerobic conditions (O_2_ < 1%) by cultivating *Lactobacillus* in jars containing AnaeroPouch (MGC, Japan).

### Cell lines

HEK293T (CCTCC, GDC187), human cervical cancer cells HeLa (ATCC, CCL-2), and human keratinocyte cells HaCaT (FuHeng, FH0186) were cultured in Dulbecco’s modified Eagle’s medium (DMEM) with 6% fetal bovine serum (FBS) (Gibco, Brazil) and 1% penicillin-streptomycin (Gibco-BRL). HUVEC (ATCC, CRL-1730) and RAW264.7 (ATCC, TIB-71) cells were cultured in DMEM with 10% FBS and 1% penicillin-streptomycin. Human THP-1 (ATCC, TIB-202) cells were maintained in Roswell Park Memorial Institute (RPMI) 1640 medium supplemented with 10% FBS and 1% penicillin-streptomycin. To induce differentiation of monocytes to macrophages, THP-1 cells were treated with 100 ng/mL PMA (Sigma-Aldrich) or 50 µg/mL nLEVs for 48 h. All cells were incubated in a humidified environment containing 5% CO_2_ at 37°C and used in the experiments, which had undergone fewer than 10 passages.

### Animals

All 7-week-old female BALB/c mice were acquired from Charles River. Mice were allowed to adapt to the new environment for a week and were maintained in SPF conditions.

### Isolation and characterization of nLEVs

nLEVs were isolated using a sequential ultracentrifugation method. Initially, a *Lactobacillus* suspension was centrifuged at 5,000 × *g* for 10 min at 4°C. The supernatant was then filtered through 0.22 µm filters to eliminate any remaining bacteria. To extract the nLEVs, the filtered supernatant underwent ultracentrifugation at 170,000 × *g* for 90 min at 4°C using a BECKMAN Optima centrifuge (plate number: XPN-100, Rotor: SW32 with a capacity of 6 × 38.5 mL). The pellet containing the nLEVs was washed with PBS, subjected to ultracentrifugation again under the same conditions, and finally resuspended in 1 mL of PBS before being stored at −80°C. The same protocol was applied to isolate particles from MRS broth. nLEV morphology was examined using transmission electron microscopy following the manufacturer’s instruction (Fei Talos, F200C, Thermo), while the size distribution and concentration were measured using a Flow NanoAnalyzer (Nanofcm), according to previously established protocols. The Zetasizer Nano ZS (Malvern Instruments Ltd, Malvern, UK) was used to determine the zeta potential of the EVs.

### Metabolite detection by LC-MS/MS analysis

The sample stored in a refrigerator at −80°C was thawed on ice. A 500 µL solution (methanol:water = 4:1, vol/vol) containing the internal standard was added into the sample and vortexed for 3 min. The sample was placed in liquid nitrogen for 5 min, then thawed on ice, and vortexed for 2 min. This freeze-thaw cycle was repeated three times in total. The sample was centrifuged at 12,000 rpm for 10 min (4°C). A 450 µL of the supernatant was transferred and concentrated. A 100 µL solution (methanol:water = 7:3, vol/vol) was used to reconstitute the sample. Then, the sample was vortexed for 3 min and sonicated for 10 min in an ice bath. The sample was centrifuged at 12,000 rpm for 3 min (4°C). Aliquots of 80 µL of the supernatant were transferred for LC-MS analysis. The data acquisition was operated using the information-dependent acquisition (IDA) mode using Analyst TF 1.7.1 Software (Sciex, Concord, ON, Canada). All experiments were performed by the MetWare company. For further analysis, differential metabolites were determined by variable importance in projection (VIP, VIP >1) and *P*-value (*P*-value < 0.05, Student’s *t* test). VIP values were extracted from the OPLS-DA result, which also contain score plots and permutation plots, and generated using R package MetaboAnalystR. The data were log-transformed (log) and mean-centered before OPLS-DA. In order to avoid overfitting, a permutation test (200 permutations) was performed.

### Cytotoxicity assay *in vitro*

A total of 10,000 cells were seeded in a 96-well plate with 100 µL of medium and incubated with nLEVs at different concentrations (5 µg, 10 µg, and 50 µg per mL). The cell viability of the cells after treatment for 48 h was measured by the CCK-8 assay to evaluate the cytotoxicity of nLEVs according to the manufacturer’s protocol (CK001, Lablead).

### Wound healing assay

A culture insert was fixed in a 12-well plate, and 50 µL of the cell suspension was seeded into each chamber of the insert following cell counting. After the cells reached confluence, the insert was carefully removed with forceps to generate a uniform scratch. The monolayer was washed with PBS to remove the debris, and the initial scratch (0 h) was imaged under a microscope. Subsequently, the scratches were treated with PBS, 50 µg/mL nCEV, 50 µg/mL nJEV, or 50 µg/mL nIEV for 24 h. Following PBS washes, images were captured again, and scratch closure was quantified using ImageJ.

### Tube formation assay

HUVEC cells were serum-starved for 12 h prior to the experiment. Precooled pipette tips and Matrigel (10 mg/mL) were prepared, and a 24-well plate was pre-warmed at 37°C. Each well was coated with 100 µL Matrigel, avoiding bubbles, and washed with PBS to prevent drying. After 30 min of incubation at 37°C, HUVEC cells were seeded and treated with PBS, 50 µg/mL nCEV, 50 µg/mL nJEV, or 50 µg/mL nIEV. Tube structures were imaged using a microscope following 6–8 h of incubation and analyzed with ImageJ.

### FITC-dextran permeability assay

Transwell chambers were pre-equilibrated in serum-free DMEM at 37°C for 20 min. HaCaT cells were seeded into the upper chamber, with 500 µL DMEM in the lower chamber, and cultured to confluence. Cells were treated with PBS, 50 µg/mL nCEV, 50 µg/mL nJEV, or 50 µg/mL nIEV for 24 h, washed with PBS, and incubated with fresh 20 µg/mL FITC-dextran (light-protected) in the upper chamber and PBS in the lower chamber for 1 h under dark conditions. Fluorescence intensity in the lower chamber was measured at 492/520 nm (excitation/emission) using a microplate reader.

### Immunoblotting assay

Total proteins were extracted either from nLEVs or from cells, using RIPA lysis (150 mM NaCl, 50 mM Tris-HCl [pH = 7.5], 2 mM EDTA, 1% NP-40) containing protease inhibitors. We loaded 50 µg of proteins on a 10% gel (Epizyme), separated them using SDS-PAGE, and then transferred them to a nitrocellulose membrane and probed them with anti-TSG101 (sc-101254, Santa Cruz), primary anti-mouse monoclonal antibodies, and then goat peroxidase-conjugated anti-mouse IgG secondary antibody. The immunoblots were detected with an Odyssey Infrared Imaging System (Li-Cor Biosciences). For Coomassie blue staining, the gel was stained with 0.1% Coomassie Blue R250 solution, including 10% acetic acid, 25% isopropanol, and 65% H_2_O, for 1 hour and then washed with ddH_2_O overnight.

### Immunofluorescence

Cells were plated on coverslips, and the glass coverslips of cells were fixed with 4% paraformaldehyde at room temperature for 30 min and blocked with PBS containing 3% BSA and 0.5% Triton X-100 for 10 min. To examine the phagocytosis of cells, nLEVs were labeled with PHK67 (Yeasen) for 10  min and washed with PBS and then collected by centrifugation at 170,000 × *g* for 90  min. Freshly stained nLEVs were added to the cells at different time points (2 h, 6 h, and 24 h). Labeling of cytoskeletal F-actin was done with rhodamine phalloidin (Yeasen). To evaluate intracellular HPV16 PsV, cells were incubated with an anti-HPV16 L1 antibody (NB100-2732, Novusbio) for 2 h at room temperature. Then, cells were incubated with a secondary antibody (goat & Rabbit 594), and the nuclei were stained with 0.5 µg/mL DAPI (Solarbio) for 3 min and sealed with a mounting medium and visualized using Leica SP8 confocal microscopy.

### Enzyme-linked immunosorbent assay (ELISA)

Fresh skin wound samples were collected and mixed with RIPA buffer containing a protease inhibitor while kept on ice to maintain temperature. Then, the supernatant was collected by spinning the samples at 12,000 rpm for 10 min at 4°C. Protein concentrations in the liquid layer above the skin tissue and the supernatants of RAW264.7 cells were measured using ELISA kits designed for mouse IL-10, TNF-α, IL-6, and TGF-β (Biotechwell Company).

### Flow cytometry

For the examination of surface markers, cells were labeled with PE-Cy7 rat anti-human CD11b (BioLegend, 552850) and PE mouse anti-human CD86 (BioLegend, 557344) antibodies for 30 min on ice in dark. Examining intracellular markers involves fixation and permeabilization for 20 min and labeling cells with BV421 mouse anti-human CD206 (BioLegend, 564062). After being washed twice, the cells were then analyzed using a flow cytometer (Cytomics FC 500, Beckman-Coulter).

### ROS measurement

Cells were plated at 5 × 10^4^ cells/mL in 96-well plates and treated with nLEVs (with or without LPS/IFN-γ) for 48 h. ROS levels were measured with a bioluminescent kit (Beyotime, China) for ROS quantification, following the provided guidelines.

### Quantitative real-time PCR

Total RNA was extracted from cells using an EZ-press RNA Purification Kit (EZBioscience). Then, qPCR was performed using TB Green Premix Ex Taq II (Takara) in a CFX96 Real-Time PCR Detection System (BioRad). According to the manufacturer^’^s protocol, PCR conditions were performed using the following steps: 1 cycle at 95°C for 5 min, followed by 40 cycles of 95°C for 15 s, 60°C for 30 s, and 72°C for 30 s. A melting-curve analysis was performed to verify the specificities of the amplified products. Relative mRNA expression levels were calculated according to ΔΔCt upon normalization to GAPDH expression, and samples were tested in triplicates.

### Cutaneous wound healing mouse model

In a BALB/c mouse, an 8 mm circular wound was created in the full thickness of the back after shaving and sterile cleaning of the area. Four groups were formed with five mice in each group, and they were treated locally with PBS or nLEVs. Following the surgery, the wound was uniformly dressed with surgical bandages (3 m, 9546) and the bandages were then removed. The wound sizes were photographed and measured with ImageJ at the indicated time points (days 0, 4, 6, 8, 10, and 12). Mice were euthanized 12 days after the surgery, and the damaged regions were harvested for additional examination.

### Production of HPV16 pseudoviruses

HPV16 pseudoviruses (PsVs) were produced, as described previously. 293T cells were seeded in a 10 cm dish the day before transfection to reach 60% cell density. Then, the cells were transfected with a mixture of p16sheLL (#37320, Addgene) and pCLucf (#37328, Addgene) plasmid using PEI (Solarbio). The cells were resuspended in lysis buffer and incubated for 24 h at 37°C. The lysate was cooled on ice for 10 min and then centrifuged at 5,000 rpm for 10 min at 4°C. Then, PsVs were purified by ultracentrifugation (4 h, 50,000 rpm, 16°C in an SW41 rotor) through an OptiPrep Density Gradient Medium (Sigma). The gradient fraction of pseudovirion stocks was stored at −80°C in siliconized microcentrifuge tubes.

### HPV inhibition assay

HaCaT cells were seeded at 5 × 10^5^ cells/well on a 6-well plate 24 h before infection. HPV16 PsVs (equivalent to 2 µg per mL) and various concentrations of nLEVs (5, 10, 50, and 100 µg per mL) were then added to the cells and incubated at 37°C for 24 h. After washing with PBS, cells were maintained in DMEM with 1% FBS for an additional 72 h. Then, cells were lysed, and the infectivity was measured by quantitative luciferase assay, according to the manufacturer’s manual (Promega).

### HPV16 pseudoviruses challenge mouse model

The establishment of a genital HPV PsV infection model was performed as described previously. Briefly, all mice received 3 mg of medroxyprogesterone acetate (MCE) in a subcutaneous injection on day 0. For the vaginal challenge, the mice were intravaginally instilled with nonoxynol-9 (N-9) (Selleckat) on day 4. Six hours later, the mixture of HPV16 PsV and 4% carboxymethyl cellulose (CMC) (Sigma) was inoculated by a cytobrush after mechanical disruption of the genital tract. Mice were pretreated intravaginally with nLEVs before PsV infection. After 72 h, *in vivo* luciferase expression was measured, according to the manufacturer’s protocols (Yeasen), and imaging was performed using an IVIS spectrum imaging system.

### Quantification and statistical analysis

All statistical analyses were performed with GraphPad Prism 8.0.2 (GraphPad). All data were expressed as the means ± standard deviation (SD). Statistical comparisons between data sets were performed by analyzing normality and variance. Student’s *t*-tests (two-tailed) were used to calculate statistical significance between the two groups. Analysis of variance (ANOVA) was utilized to compare differences among multiple groups. In all experimental sections, unless otherwise stated, the *t*-test was used for statistical analysis. At the same time, Tukey’s *post hoc* test was applied for further comparisons to assess the significance of differences between the groups following the one-way ANOVA. *P* values < 0.05 were considered statistically significant.

## RESULTS

### Optimized production of nLEVs derived from *Lactobacillus*

**Fig 1:**
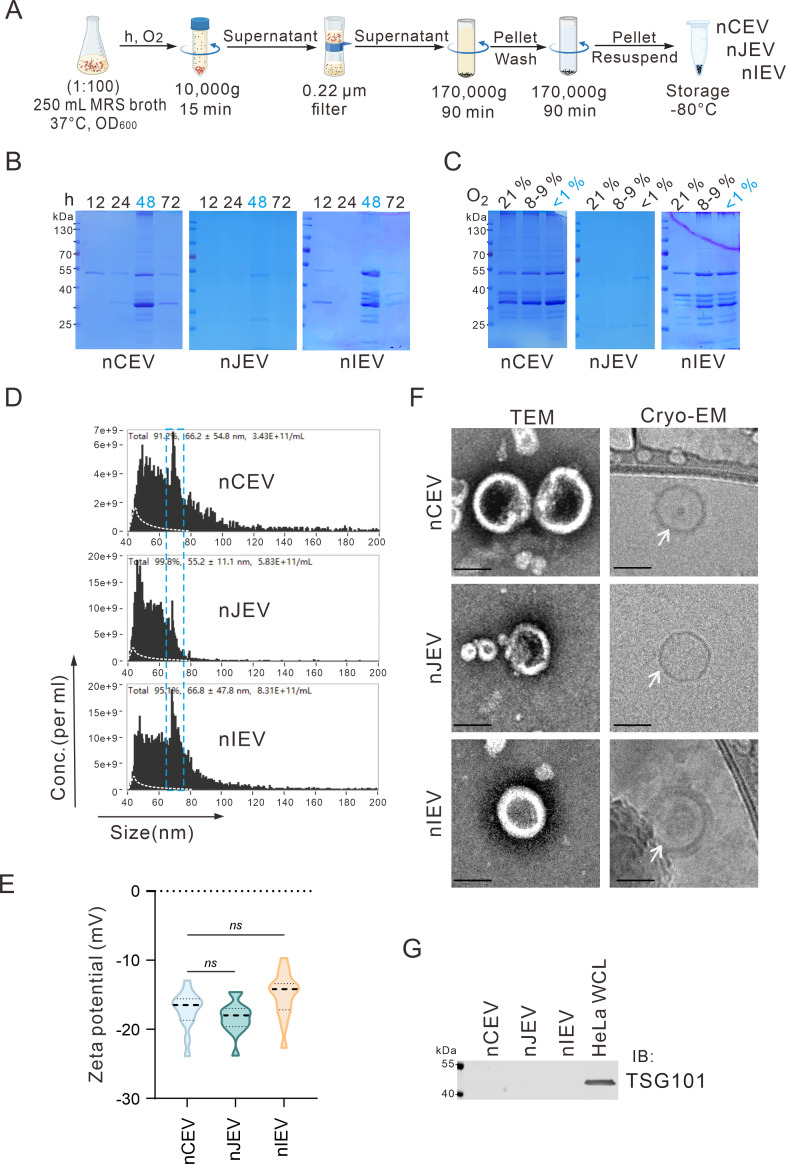
Isolation and characterization of nLEVs from *Lactobacillus*. (A) Schematics of 60–80 nm size dominance of nLEV vesicle isolation from *Lactobacillus*. (B) Coomassie blue staining shows the production of nLEVs obtained from *Lactobacillus* MRS broth at different cultivation times. (C) Coomassie blue staining shows the production of nLEVs obtained from *Lactobacillus* MRS broth at different oxygen culture (21%, 8%–9%, and <1%). (D) The particle size and concentration of nLEVs using Flow NanoAnalyzer analyses. (E) The zeta potentials of nLEVs (*n* = 15). (F) TEM and Cryo-EM image analyses of the structure of nLEVs. All scale bars are 50 nm. White arrowheads indicate the double membrane of the nLEVs. (G) Comparison of nLEVs with mammalian EVs by immunoblotting analysis. WCL, whole-cell lysate.

For substantial production of LEVs from *Lactobacillus* MRS broth, we explored the optimal conditions by changing bacteria incubation time and oxygen levels and adjusting centrifugal speed and time, as described in the procedure in Fig. 1A. The results showed that all three *Lactobacillus* strains (*L. crispatus, L. jensenii*, and *L. iners*) produced the highest number of nLEVs (nCEVs, nJEVs, and nIEVs) when incubated for 48 h in an anaerobic environment (O_2_ < 1%) (Fig. 1B and C). Notably, we found the particle sizes of the three EVs present a similar dominant peak in sizes between 60 nm and 80 nm, although the mean sizes of total nCEV, nJEV, and nIEV were slightly varied from 66.2 nm (for nCEV), 55.2 nm (for nJEV), to 66.8 nm (for nIEV) (Fig. 1D), and the concentration of three nLEVs was 3.43E+11 per mL (for nCEV), 5.83E+11 per mL (for nJEV), and 8.31E+11 per mL (for nIEV), respectively (Fig. 1D). In contrast, the MRS broth, not conditioned by bacteria, also contained particles with approximately 2.11E+10 per mL. The average zeta potential values of nCEV, nJEV, and nIEV were measured as −17.17 mV, −18.29 mV, and −15.03 mV, respectively (Fig. 1E). This negative surface charge characteristic of LEVs aligns with previous reports in the field. Notably, the values approaching −20 mV demonstrate favorable colloidal stability of these nanovesicles. The morphology of nLEVs examined by using transmission electron microscopy (TEM) and cryo-transmission electron microscopy (cryo-EM) (Fig. 1F) showed that the three nLEVs exhibited a closed membrane structure, akin to previously documented bEVs derived from gram-positive bacteria (27), which were spherical and encased in a lipid bilayer (Fig. 1F) and different from eukaryotic EV with the TSG101 marker (Fig. 1G).

### nCEVs enhance cell migration, angiogenesis, and barrier integrity *in vitro*

**Fig 2:**
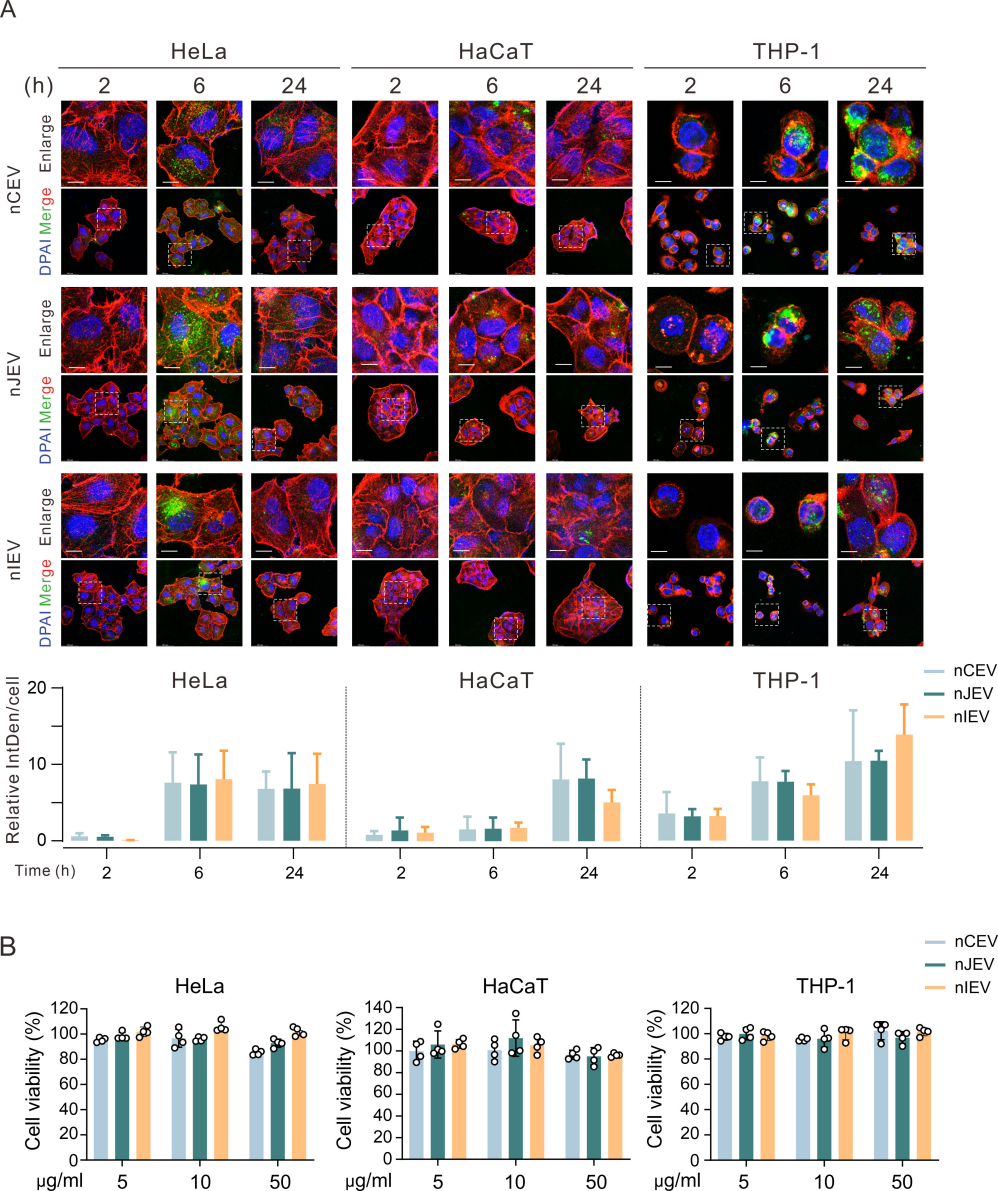
nLEVs can be internalized and show no cytotoxic effects. (**A**) nLEVs were labeled with PKH67 and observed in the cytoplasm of HaCaT, HeLa, and THP-1 cells after incubation for 2, 6, and 24 h. Cells were stained with TRITC-phalloidin and DAPI. Quantitative analysis of cellular uptake was performed by measuring the relative fluorescence intensity per cell (three independent fields per sample). IntDen: integrated option density. Scale bars are 10 and 36.8 µm. (**B**) Cell viability according to CCK-8 assay of HaCaT, HeLa, and THP-1 cells after 48 h of incubation (*n* = 4).

To investigate the cellular internalization potential of nLEVs, we evaluated their uptake by HeLa, HaCaT, and THP-1 cell lines using confocal microscopy. PHK67-labeled nLEVs were tracked at 2, 6, and 24 h incubation intervals to assess temporal uptake patterns. Microscopic analysis revealed characteristic punctate signals accompanied by diffuse cytoplasmic fluorescence in all cell types after 6 h of co-incubation (Fig. 2A), suggesting both vesicular internalization and potential membrane fusion events. While no statistically significant differences in uptake efficiency were observed among the three nLEV subtypes, distinct cell-type-dependent variations emerged. Notably, THP-1 cells demonstrated substantially higher nLEVs accumulation over 24 h compared to HaCaT cells. Furthermore, HeLa cells exhibited enhanced uptake relative to HaCaT cells at the 6 h time point (Fig. 2A). Our experimental focus was on vaginal epithelial physiology: while HeLa cells demonstrated higher baseline uptake efficiency, we used HaCaT keratinocytes—a validated squamous epithelial model—to better recapitulate the native vaginal epithelial microenvironment. These findings indicate that nLEVs can be internalized by cells. To further determine whether nLEVs treatment influences cell viability, we examined the potential cytotoxic effects of each nLEV on HeLa, HaCaT, and THP-1 cells in different dosages by using CCK-8 assay. The results showed no significant reduction in the three types of cells’ viability after treatment with different concentrations of nLEVs (Fig. 2B), suggesting that the three nLEVs do not exert cytotoxic effects.

**Fig 3:**
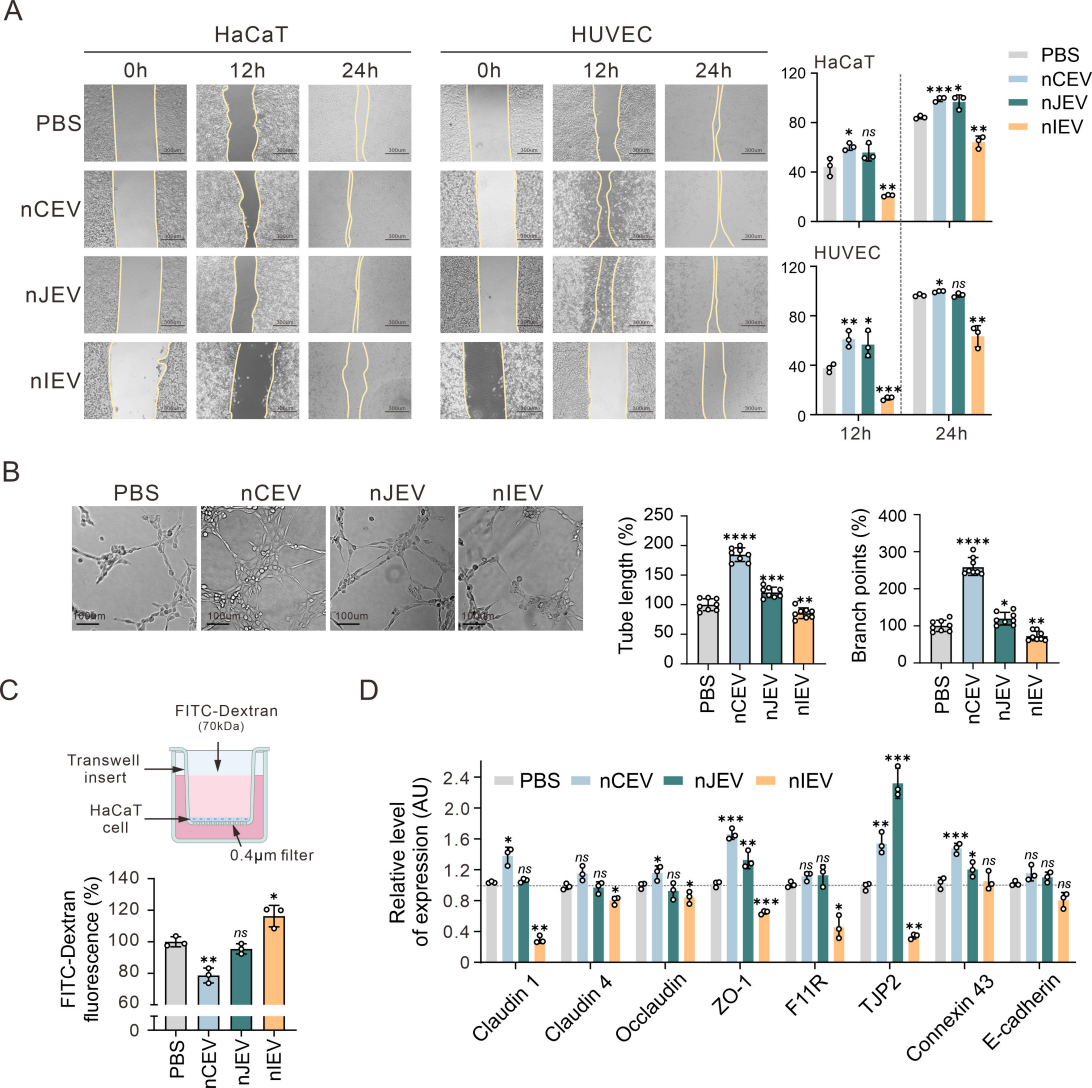
nCEVs induce cell migration and angiogenesis *in vitro*. (**A**) The scratch wound healing assay investigated the migratory ability of HaCaT and HUVEC after exposure to nLEVs or PBS for 24 h, respectively (*n* = 3). All scale bars are 300 µm. The percentage of healing rate is shown at the right panels. (**B**) Tube formation of HUVECs after exposure to nLEVs or PBS for 6 h (*n* = 8). All scale bars are 400 µm. (**C**) FITC-dextran transepithelial permeability assay to quantify the permeability of the HaCaT monolayer after exposure to nLEVs or PBS for 24 h (*n* = 3). (**D**) Quantitative PCR analysis of the gene expression of cell junction-related molecules in HaCaT cells after exposure to nLEVs or PBS for 24 h (*n* = 3). *P < 0.05; **P < 0.01; ***P < 0.001; ****P < 0.0001; *ns*, not significant.

Given the capacity of cells to rapidly internalize nLEVs, to investigate the effects of nLEVs on cell migration, we analyzed two cell types of HaCaT and HUVEC by using scratch-wound assays. The results showed that both nCEV and nJEV treatments in HaCaT and HUVEC cells significantly enhanced cell migration compared to the control group, while treatment with nIEV resulted in a reduced migration rate (Fig. 3A). To further investigate the influence of nLEVs on angiogenesis, we evaluated their effects on HUVEC tube formation. The results showed that HUVEC cells in both nCEV and nJEV group, particularly in the nCEV group, were capable of forming a mesh-like tubular structure after 6 h co-incubation (Fig. 3B). In contrast, no significant angiogenesis was observed in the control and nIEV groups, indicating that both nCEV and nJEV may enhance cell angiogenesis. Given previous observations of a link between vaginal *Lactobacillus* and the integrity of cervicovaginal epithelial cells (43), we further investigated the effects of nLEVs on epithelial barrier function by using the FITC-dextran leakage assay. The results showed that stimulation with nIEV resulted in an increased flux of FITC-labeled dextran in HaCaT cells, indicating compromised barrier integrity. In contrast, treatment with other nLEVs, particularly nCEV, significantly reduced the leakage of FITC-dextran (Fig. 3C). To further elucidate the potential mechanisms by which nLEVs influence barrier function, we determined the expression level of genes that encode critical junctional proteins in HaCaT cells treated with nLEVs. The results showed a significant increase in the expression of occludin, ZO-1, and TJP2 after nCEV treatment, while nIEV treatment resulted in a decrease in the expression of these genes (Fig. 3D). Collectively, these findings indicate that nCEV enhances the functionality of keratinocytes and endothelial cells, whereas nIEV exerts the opposite effect.

### nCEVs induce skin wound healing *in vivo*

**Fig 4:**
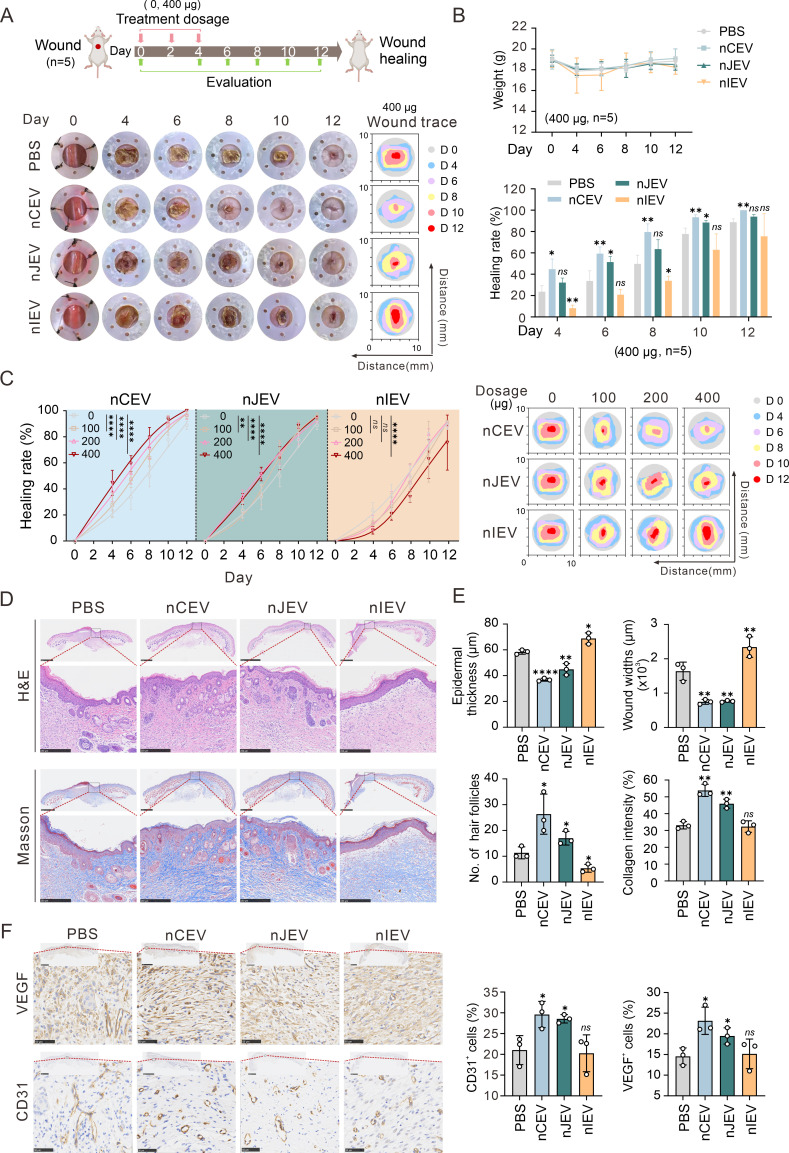
nCEVs enhance wound healing *in vivo*. (**A**) A brief flowchart of this experiment and representative images of skin injuries and corresponding wound trace images in different groups (PBS, nCEV, nJEV, and nIEV) at different time points during the wound healing procedure. The dosage of 400 µg in 50 µL nLEVs was used. The equivalent volume of PBS was used as a control. (**B**) Quantitation of the wound healing rate and the body weight of mice (two-way ANOVA tests, *n* = 5). (**C**) Representative wound trace images using different dosages (100 µg, 200 µg, or 400 µg in 50 µL) of nLEVs (nCEV, nJEV, and nIEV) at different time points during the wound healing procedure (*n* = 5). (**D**) Representative images of the H&E and Masson staining of the skin samples. Scale bars are 1 mm in low-magnification images and 250 µm in high-magnification images. (**E**) Quantitation of epidermal thickness, wound width, number of hair follicles, and collagen intensity in the wound tissue (*n* = 3) from panel A. (**F**) Representative images and quantitation of the percentage of CD31^+^ and VEGF^+^ cells in the skin tissue (*n* = 3). Scale bars are 1 mm in low-magnification images and 250 µm in high-magnification images. Data are shown as mean ± SD. *P < 0.05; **P < 0.01; ***P < 0.001; ****P < 0.0001; *ns*, not significant.

To investigate the impact of nLEVs on wound healing, we used a BALB/c mouse model with full-thickness cutaneous wounds measuring approximately 8 mm in diameter. The mice were divided into four treatment groups of nCEV, nJEV, and nIEV, along with PBS as a control, followed by treatment with nLEVs or PBS injected apically into the wound margins twice a day for three times (Fig. 4A, top panel). The results showed that wounds treated with nCEV or nJEV exhibited significant contraction compared to those treated with PBS and nIEV on day 4 post-injury. On day 12, the nCEV group showed complete wound closure, while the wound area in the nJEV group decreased by approximately 93.9%. In contrast, wounds in the PBS and nIEV groups showed reductions of about 88.7% and 75.6%, respectively (Fig. 4A and B, bottom panels). There were no significant differences in the body weights of the mice across the four groups (Fig. 4B, top panel). To further examine the effect of nLEVs treatment on wound healing, we administered different dosages of nLEVs to treat the wounds in the mice. The results of the wound trace showed that higher dosages of nCEV and nJEV, but not nIEV, resulted in a more pronounced effect on the wound healing rate (Fig. 4C).

To further confirm the wound healing process, we conducted a series of pathological tests using hematoxylin and eosin (H&E) staining as well as Masson’s trichrome staining. The results showed that the wound was nearly invisible on day 12 in both the nCEV and nJEV groups, while the nIEV group exhibited a larger wound compared to the PBS group (Fig. 4D, upper panels) and presented a significantly higher level of collagen deposition in the nCEV group, whereas the nIEV group exhibited the lowest levels (Fig. 4D, lower panels), indicating a remarkable enhancement in cutaneous wound healing with nCEV treatment across the four groups. Further analysis revealed that the nCEV group significantly improved integration of skin tissue with the regenerated epithelium, characterized by an increased formation of hair follicles, better organization, and greater collagen deposition, albeit the nJEV group also displayed a notable reduction in scar size, along with improved organization and deposition of collagen fibers (Fig. 4E). Conversely, compared to the PBS group, nIEV treatment resulted in an increase in scar size and a reduction in the organization and deposition of collagen fibers. These findings suggest that nCEV treatment may be most effective in accelerating skin wound healing and improving the quality of skin regeneration. Given vascular endothelial growth factor (VEGF) and CD31 have been demonstrated to contribute to wound tissue neovascularization, we also determined the expressions of CD31 and VEGF in wound tissues treated with each nLEV by immunohistochemical staining and found that the nCEV group indeed had significantly higher proportions of CD31- and VEGF-positive cells in wound tissue than the nJEV and nIEV groups, suggesting that nCEV could induce neovascularization in wound healing (Fig. 4F).

### nCEVs can induce macrophage activation and M2 polarization

**Fig 5:**
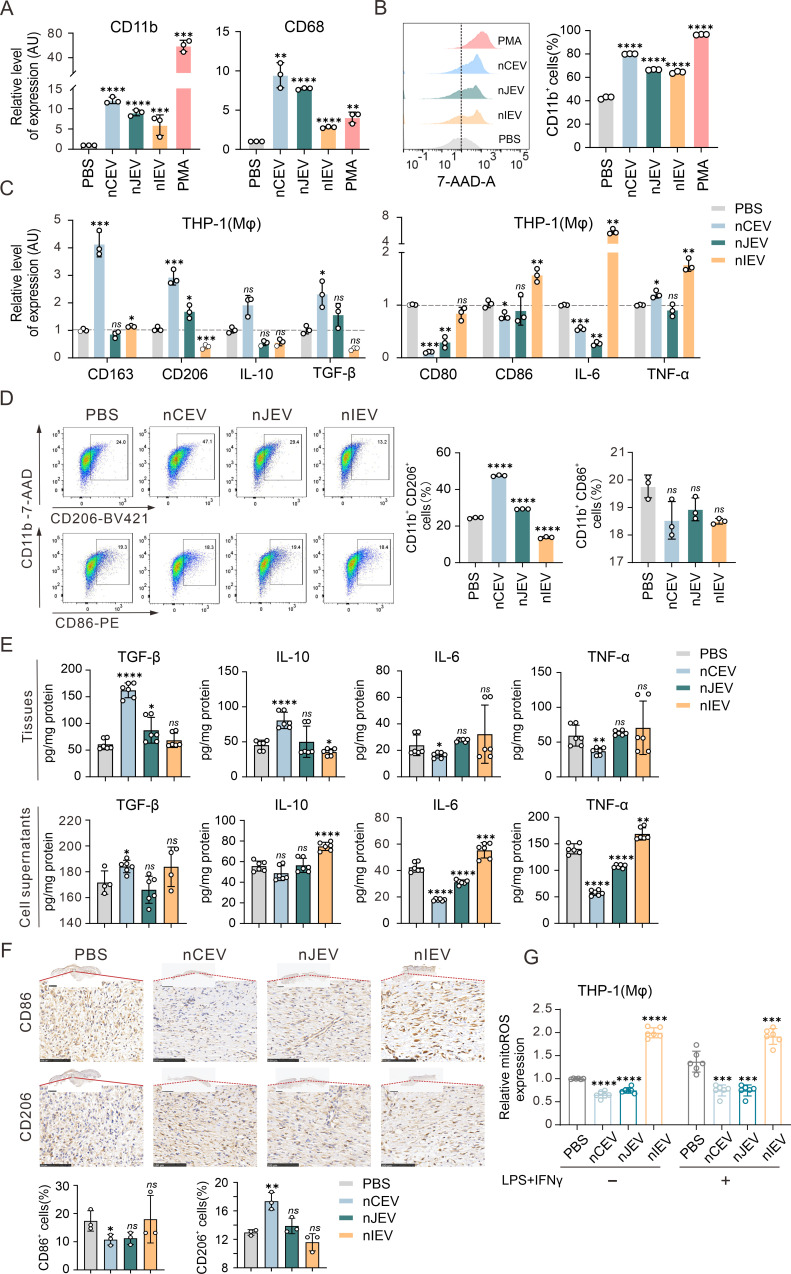
nCEVs activate monocyte and elicit M2 polarization of macrophages. (**A**) The mRNA expressions of CD68 and CD11b (activated macrophage-specific genes) in THP-1 cells treated with nLEVs or PBS for 48 h by quantitative PCR analysis. THP-1 cells treated with 100 ng/mL PMA for 48 h were used as a positive control. (**B**) The expression level of CD11b in THP-1 cells treated with nLEVs or PBS for 48 h was analyzed by flow cytometry. THP-1 cells treated with 100 ng/mL PMA for 48 h were used as a positive control. (**C**) The mRNA expression of typical M2 markers (CD163, CD206, TGF-β, and IL-10) and M1 markers (CD80, CD86, IL-6, and TNF-α) in PMA-pretreated THP-1 cells treated with nLEVs or PBS for 48 h was analyzed by quantitative PCR. (**D**) Flow cytometry analysis of the expressions of CD206 and CD86 in PMA-pretreated THP-1 cells treated with nLEVs or PBS for 48 h. (**E**) Cytokine expression (TGF-β, IL-10, IL-6, and TNF-α) in cutaneous wounds from Fig. 3A and the supernatants of RAW264.7 cells treated with nLEVs or PBS for 48 h was analyzed by ELISA (*n* = 6). (**F**) Representative images and quantitation of the percentage of CD206^+^ and CD86^+^ cells in the skin tissue (*n* = 3). Scale bars are 1 mm in low-magnification images and 250 µm in high-magnification images. (**G**) The level of reactive oxygen species (ROS) in (100 ng/mL) PMA-pretreated THP-1 cells treated with nLEVs or PBS in the presence or absence of LPS (100 ng/mL) and IFN-γ (20 ng/mL) for 48 h. Data are shown as mean ± SD. *P < 0.05; **P < 0.01; ***P < 0.001; ****P < 0.0001; *ns*, not significant.

Given macrophages play a pivotal role in wound healing and tissue remodeling (44, 45), we hypothesized that nLEVs may impact monocyte differentiation and macrophage activation and determined the expression levels of the two key markers CD68 and CD11b in THP-1 cells treated with nLEVs. Interestingly, similar to the observation with phorbol 12-myristate 13-acetate (PMA) treatment as a positive control, we found that treatment with three nLEVs notably enhanced the expression of CD68 and CD11b compared to PBS treatment (Fig. 5A), which was further confirmed by flow cytometry analysis (Fig. 5B). These findings suggest that all three types of nLEV treatments can facilitate the differentiation of monocytic cells into the macrophage lineage, even in the absence of PMA, thereby potentially influencing the monocyte fate.

Since nLEVs can promote the differentiation of monocytes into the macrophage lineage, we attempted to determine which polarization of macrophage is induced by nLEVs by detecting the expression of M1 and M2 macrophage biomarkers at the mRNA and protein levels after nLEV treatments. The results from quantitative PCR analysis showed that treatment with nCEV and nJEV tended to decrease the mRNA expression of pro-inflammatory M1-type markers (CD80, CD86, and IL-6) while enhancing the expression of anti-inflammatory M2-type markers (CD206, CD163, IL-10, and TGF-β), albeit the levels of M2 polarization induced by nJEV were much lower than those induced by CEV (Fig. 5C). In contrast, nIEV treatment presented the opposite effects (Fig. 5C). Consistently, we observed that nCEV instead of nJEV or nIEV greatly increased the population of CD11b^+^CD206^+^ cells for M2 macrophage polarization by flow cytometry analysis and reduced CD11b^+^CD86^+^ cells for M1 macrophage polarization to some extent (Fig. 5D). Although IEV could markedly reduce the population of CD11b^+^CD206^+^ cells for M2 macrophage polarization, we did not observe the population of CD11b^+^CD86^+^ cells enhanced by nIEV treatment. The results indicate that nCEVs present a more pronounced effect on the activation of M2-type macrophages.

To further confirm the role of nCEV in wound healing, we also evaluated the levels of various inflammatory factors in wound-healing tissues and cellular supernatants by using ELISA. Notably, consistent with our previous observations, nCEV appeared to reduce the production of pro-inflammatory factors (IL-6 and TNF-α), while enhancing the secretion of anti-inflammatory cytokines (IL-10 and TGF-β) in both tissues and cell supernatants (Fig. 5E, upper panels). In contrast, the effect of both nJEV and nIEV on secretory inflammatory factors was relatively rare in wound tissues, while nIEV increased the levels of pro-inflammatory factors, except for IL-10, in cell supernatants (Fig. 5E). The results from the immunohistochemistry assays of wound tissues with CD206 and CD86 staining showed a dramatic increase in the percentage of CD206^+^ macrophages near the wound in the nCEV group (Fig. 5F), suggesting the potent role of nCEVs in promoting the activation of macrophages toward the M2 phenotype.

In view of the significant relationship between mitochondrial activity and the production of reactive oxygen species (ROS) during the phenotypic transition of macrophages, to further explore the role of these nLEVs in macrophage polarization, ROS levels were assessed in THP-1 cells with LPS/IFN-γ treatment as a positive control. As anticipated, both nCEV and nJEV exhibited an inhibitory effect on the production of ROS within the cells (Fig. 5G, left panel). Although the introduction of LPS/IFN-γ enhanced ROS levels in THP-1 cells, it did not impair the inhibitory effects of both nCEV and nJEV, while nIEV significantly enhanced ROS levels, irrespective of the presence of LPS/IFN-γ (Fig. 5G). The findings indicate that nCEV has a significant effect in promoting the polarization of macrophages toward the M2 phenotype for wound healing.

### nCEVs block the infection of HPV16 *in vitro* and *in vivo*

**Fig 6:**
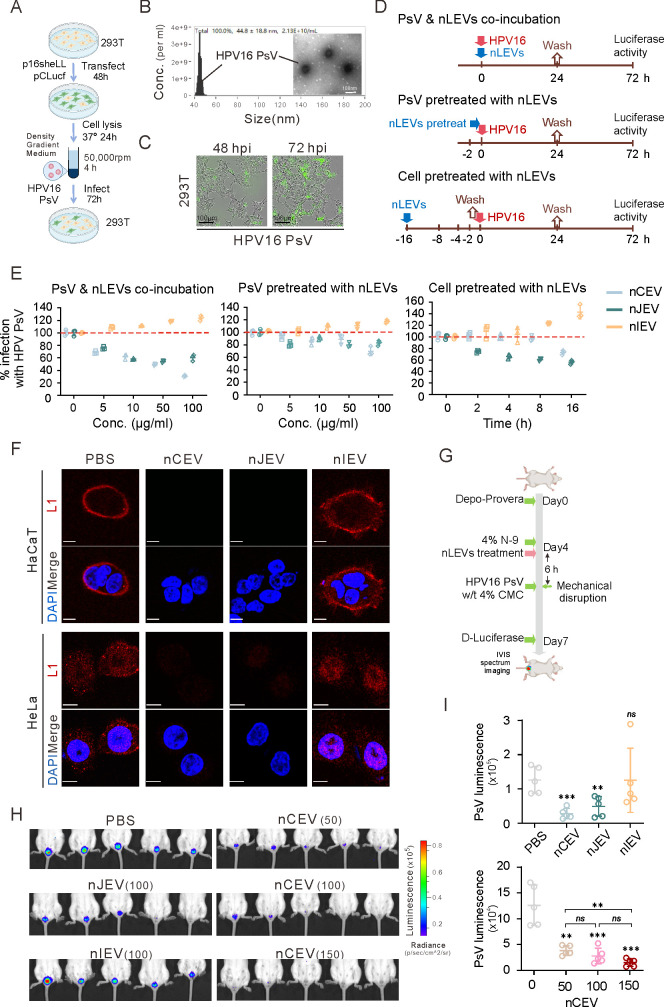
nCEV inhibit the infection of HPV16 *in vitro* and *in vivo*. (**A**) The flowchart of HPV16 PsV construction. (**B**) The size and concentration of the HPV16 PsV were analyzed using a Flow NanoAnalyzer and TEM. Scale bar is 100 nm. (**C**) Representative images of 293T infected with HPV16 PsV for 48 and 72 h. All scale bars are 100 µm. (**D**) Schematics of PsV and nLEVs co-incubation assay, PsV pretreated with nLEVs assay and cells pretreated with the nLEVs assay. (**E**) The percentage of HPV16 inhibition by nLEVs. In the PsV and nLEVs co-incubation assay, infected HaCaT cells were treated for 24 h with different concentrations of nLEVs. The infection rate of HPV16 was calculated by comparing treated and untreated cells by luciferase assay. In PsV pretreated with nLEVs assay, HPV16 PsV was incubated with different concentrations of nLEVs for 2 h and then separated by ultrafiltration before being added to HaCaT. The infection rate of HPV16 was calculated by comparing treated and untreated cells by luciferase assay. In the cells pretreated with the nLEVs assay, HaCaT cells were treated with nLEVs for different periods and then washed with PBS to remove unabsorbed nLEVs before inoculating the cells with HPV16 PsV. The infection rate of HPV16 was calculated by comparing treated and untreated cells by luciferase assay. (**F**) Representative immunofluorescence images of HeLa and HaCaT cells infected with HPV16 PsV in the absence or presence of nLEVs. Cells were stained with anti-HPV16 L1 antibody and DAPI for nuclear compartment. Scale bar is 10 mm. (**G**) Schematics of HPV16 PsV challenge mouse model construction. (**H, I**) Mice were challenged intravaginally with HPV16 PsV and treated with nCEV (50 µg, 100 µg, and 150 µg in 30 µL), nJEV (100 µg in 30 µL), nIEV (100 µg in 30 µL), or 30 µL PBS. Bioluminescence was visualized using a Xenogen camera. Quantitation of the infection rate of HPV16. Bioluminescence was measured in p/sec/cm^2 /sr. Data were shown as mean ± SD. *P < 0.05; **P < 0.01; ***P < 0.001; ****P < 0.0001; *ns*, not significant.

HPV16 is recognized as a high-risk type that causes cervical cancer (46). To investigate the potential effects of nLEVs on HPV16 infection, we generated and harvested HPV16 pseudovirus particles (PsV) from 293T cells with co-transfection of p16sheLL and pCLucf plasmids (Fig. 6A). The results from flow NanoAnalyzer and TEM microscopy analysis showed that PsV is a non-enveloped virus with a diameter of 40 to 55 nm (Fig. 6B). Subsequently, we infected 293T cells with this PsV and observed that the maximum infection rate was reached after 72 h (Fig. 6C). To fully evaluate the role of nLEVs on the infection of HPV16 PsVs, we conducted a series of experiments by co-incubation or pretreated with PsV or HaCaT cells with nLEVs, as described in Fig. 6D. The results showed that when co-incubating PsV with nLEVs, both nCEV and nJEV treatments could significantly inhibit HPV16 infection in a dose-dependent manner, while nIEV slightly increased the HPV16 infection rate along with dosage increase (Fig. 6E, left panel). When HaCaT cells were pretreated with each nLEV for different periods, followed by infection of PsVs, the results indicated that the inhibitory effects of nCEV and nJEV on HPV16 infection are in a time-dependent manner, which exhibited an inhibitory peak on HPV16 infection at 16 h (Fig. 6E, right panel). In the case that the PsVs were pretreated with nLEVs for 2 h at room temperature, followed by infecting cells with the treated PsVs, the results showed that each nLEV appeared to have a similar minimal effect on the activity of HPV16 PsVs (Fig. 6E, middle panel). These findings suggest that nLEVs mainly impair cellular biological processes, thereby influencing HPV16 infection. To further confirm the inhibitory effects of nCEV and nJEV on HPV entry, we attempted to determine the internalization of HPV16 PsVs in cells after infection by immunofluorescence analysis targeting the nuclear capsid L1 protein. The results showed that in the nCEV and nJEV groups, very few PsV virions presented intracellularly in both HeLa and HaCaT cells (Fig. 6F). In contrast, treatment with nIEV did not appear to show a significant difference compared to the control group (Fig. 6F), indicating that both nCEV and nJEV effectively inhibit the entry of HPV into epithelial cells, whereas nIEV may facilitate HPV infection.

To further answer the inhibitory effect of nLEVs on HPV infection *in vivo*, we generated murine genital infection models by treating each nLEV via perfusion into the vaginal tract of mice before the luciferase-expressing HPV16 PsV challenge and monitored genital infection by measuring bioluminescence with the IVIS imaging system (Fig. 6G). The results showed that although administration of both nCEV and nJEV dramatically inhibited murine genital HPV16 infection, nCEV presented much higher activity than nJEV, as a less amount of nCEV could achieve a similar effect to nJEV after nCEV was titrated down (Fig. 6H and I). In contrast, nIEV showed no effect on the infection rate of HPV16 (Fig. 6H and I). These findings indicate that nCEVs exhibit the most potent inhibitory activity on HPV16 infection *in vivo*.

### Distinct metabolomic profiles in nCEV, nJEV, and nIEV

**Fig 7:**
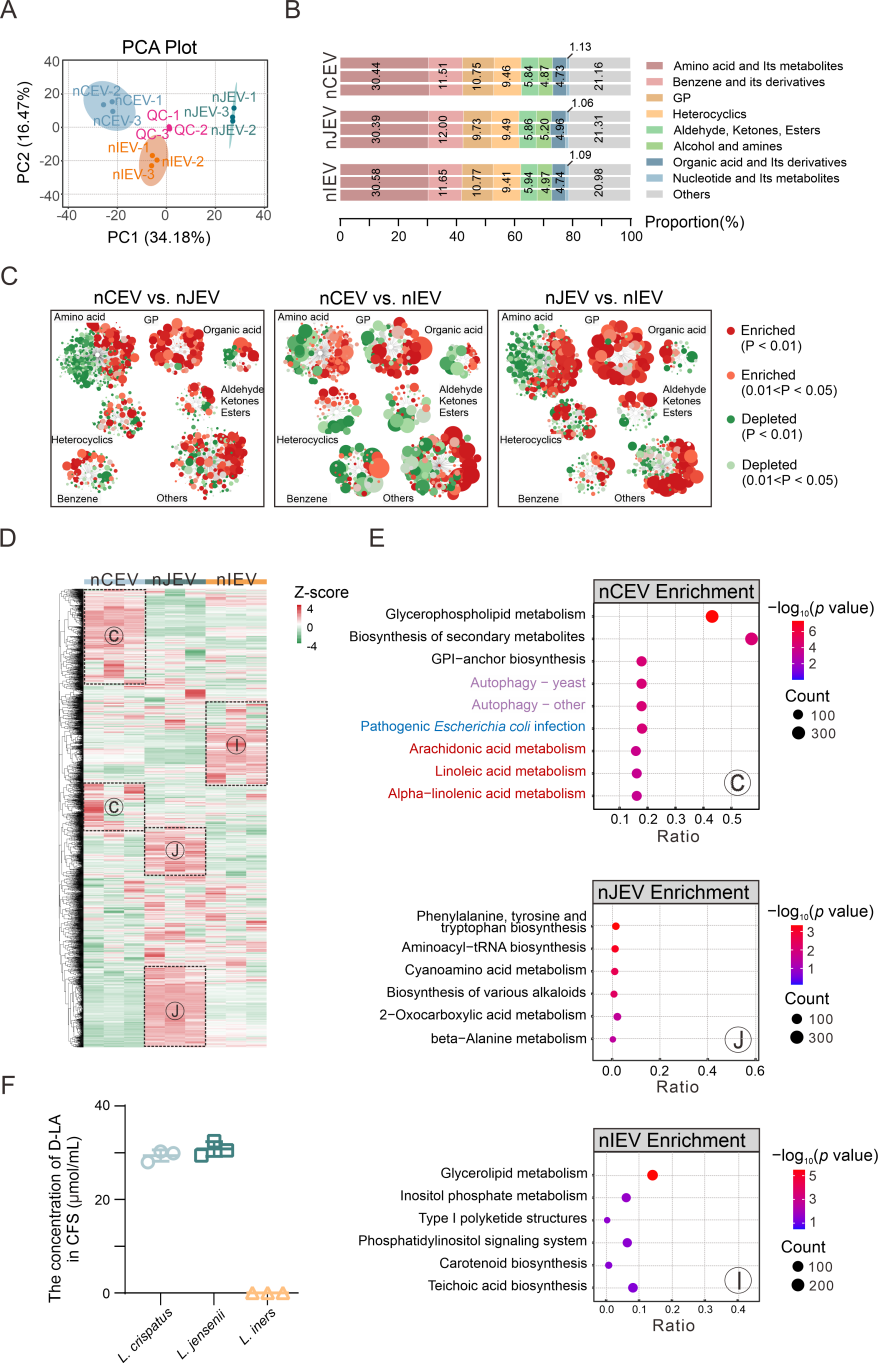
Metabolomic profiles associated with nLEVs. Principal component analysis (PCA) of three nLEVs, including quality control (QC) samples. (**B**) The classification of identified metabolites in three nLEVs. (**C**) Enrichment and depletions of metabolites among three nLEVs visualized by Cytoscape metabolic network analysis. The node size is proportional to the magnitude of differences observed among the groups. Red and green nodes represent enriched and depleted metabolites, respectively. (**D**) Heatmap clustering analysis of the significant differentially expressed metabolites (DEMs) of three nLEVs. C, nCEV; J, nJEV; I, nIEV. (**E**) KEGG pathways related to significant DEMs of three nLEVs via a bubble plot. Ratio means the ratio of the number of DEMs in the corresponding pathway to the total number of metabolites annotated to that pathway, with larger values indicating greater enrichment. Red indicates that the pathway is involved in wound healing; blue indicates that the pathway is involved in anti-infection; purple indicates that the pathway is involved in wound healing and anti-infection. (**F**) The concentration of D-LA in the cell-free probiotic supernatant (CFS) of three *Lactobacillus* MRS broth (*n* = 3).

To elucidate the difference in metabolite profiling in the nCEV, nJEV, and nIEV, we employed LC-MS/MS analysis by detecting three repeat samples and identified 2,536 metabolites across the three nLEVs. The results of the PCA score plot showed that the first principal components (PC1) effectively discriminated the metabolomic profiles of the three nLEVs, while the nLEVs from the same *Lactobacillus* strains presented similar PC scores, suggesting good biological reproducibility of the metabolomic profile (Fig. 7A). Intriguingly, we discovered that the distribution of metabolite classification was nearly identical among the three nLEVs. These metabolites predominantly included several classes, such as amino acids and their derivatives, benzene and its substituted derivatives, glycerophospholipids (GP), and heterocyclic compounds (Fig. 7B). To further define differentially expressed metabolites carried in each nLEV, we performed multivariate analysis based on their *P*-values and VIP scores, particular criteria for *P* < 0.05 and VIP >1. As shown in Fig. 7C, the metabolic network visualization of three types of nLEVs constructed using Cytoscape revealed that compared to nIEV, metabolites related to amino acid metabolism and glycerophospholipid (GP) metabolism were significantly enriched in nCEVs and nJEV. This difference may reflect the specificity of different nLEV subpopulations in terms of metabolic function regulation and mechanisms of intercellular communication. We also constructed a heatmap of the metabolic profiles with a significant difference from the three nLEVs (C for nCEV, J for nJEV, and I for nIEV) obtained (Fig. 7D). To address the functions of the major metabolites in each nLEV, we subsequently analyzed the enrichment of the KEGG pathways related to the metabolites that were highly packaged in each nLEV (Fig. 7E). Among these three nLEVs, the high-level metabolites were predominantly enriched in the pathways related to acid metabolism, amino acid metabolism, and autophagy. However, the pathways, including autophagy-yeast, pathogenic *Escherichia coli* infection, arachidonic acid metabolism, linoleic acid metabolism, and alpha-linolenic acid metabolism (which have been previously reported to be associated with tissue repair and wound healing and antimicrobial and antiviral effects of *L. crispatus* in the vaginal microenvironment [47–51]), were exclusively and significantly enriched in nCEV, but not nJEV or nIEV (Fig. 7E, top panels).

### D-lactate is a crucial component that enables nCEVs to promote wound healing and inhibit HPV16 infection

Lactic acid (LA), a metabolite generated by diverse *Lactobacillus* species, is present in two enantiomeric forms: the L- and D-isomers. This isomeric differentiation, which is species-dependent, significantly contributes to the functional heterogeneity of LA within the vaginal microbiota (52). Our experimental analysis of culture supernatants from three *Lactobacillus* revealed species-specific D-LA production patterns (Fig. 7E): both *L. crispatus* and *L. jensenii* predominantly secreted D-LA, whereas *L. iners* demonstrated negligible D-LA synthesis—a phenotypic distinction consistent with existing literature (52). Metabolomic profiling identified lactate accumulation in nLEVs, with the neutral pH microenvironment of these vesicles favoring the deprotonated lactate form over LA. While the mass spectrometry methodology employed here inherently lacks chiral discrimination capacity for enantiomeric differentiation (L-LA/D-LA), the integration of our empirical findings with established biochemical evidence strongly suggests a stereochemical partitioning: D-lactate shows enrichment in nCEV and nJEV, while its L-lactate preferentially localizes to nIEV. This stereospecific compartmentalization within vesicular organic acid metabolism implies that the lactate isomer spatial configuration may constitute an essential regulatory axis governing nLEV functional diversification. The observed chiral segregation mechanism could potentially mediate dichotomous biological effects across critical physiological processes, spanning tissue regeneration pathways to viral-host interaction dynamics.

**Fig 8:**
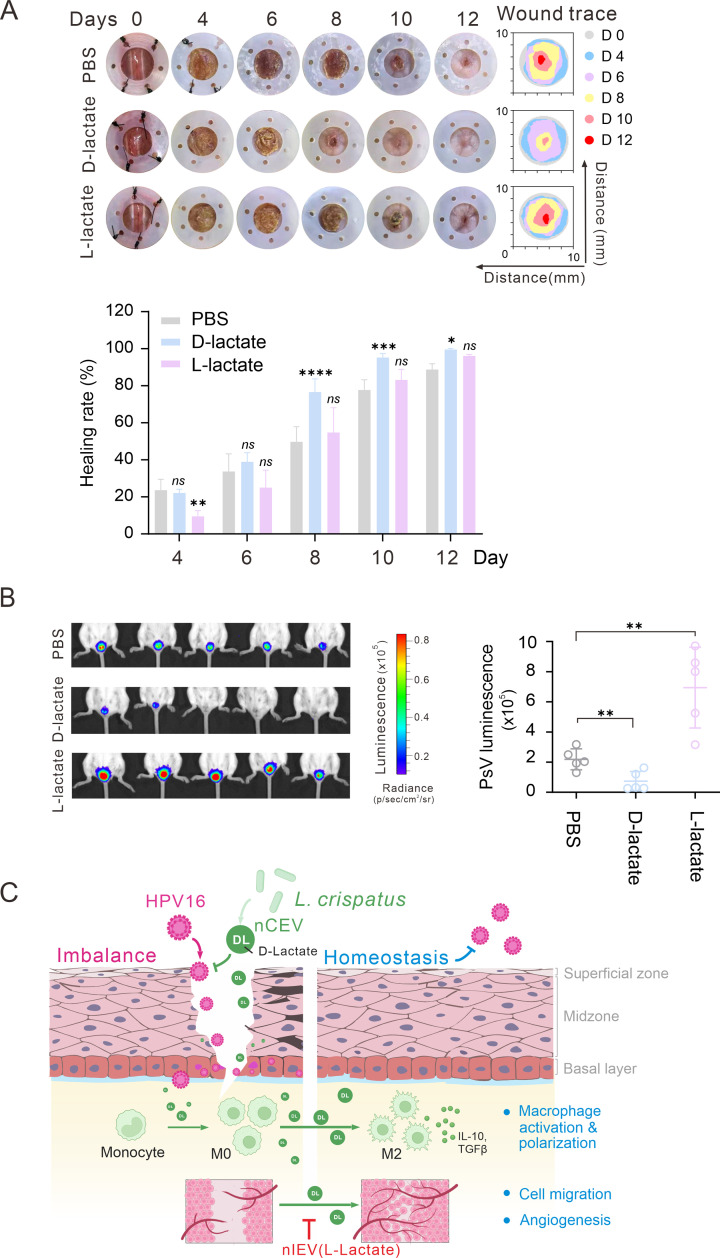
D-lactate is a key component in nCEVs for wound healing and blocking viral infection. (**A**) Representative images of skin injuries, corresponding wound trace images, and quantification of the wound healing rate in PBS (100 µL), L-lactate (100 mM/ 100 µL), and D-lactate (100 mM/ 100 µL) groups at different time points during the wound healing procedure (two-way ANOVA tests, *n* = 5). Data were shown as mean ± SD. *P < 0.05; **P < 0.01; ***P < 0.001; ****P < 0.0001; ns, not significant. (**B**) Mice were challenged intravaginally with HPV16 PsV and treated with 30 µL of PBS, L-lactate (100 mM), or D-lactate (100 mM). Bioluminescence was visualized using a Xenogen camera. Quantitation of the infection rate of HPV16. Genital bioluminescence was measured in p/sec/cm^2 /sr. (**C**) A schematic diagram shows how nCEVs (including D-lactate) instead of nIEVs (including L-lactate) promote cutaneous homeostasis for wound healing and blocking HPV16 infection via inducing macrophage M2 polarization, cell migration, and angiogenesis.

To prove this hypothesis, we performed wound healing and vaginal HPV16 infection *in vivo* assays by treating with L-lactate or D-lactate. Strikingly, the results showed that L-lactate significantly delayed the wound healing rate at the early initial period of day 4, while its impact on healing was gradually diminished when compared to the PBS control group (Fig. 8A). In contrast, the administration of D-lactate significantly promoted wound healing, which was evident after 6 days and continued until the wounds fully healed (Fig. 8A). On day 8, wounds treated with D-lactate displayed a notably smaller area compared to the PBS group, and on day 12, the wounds treated with D-lactate showed an impressive reduction of approximately 99.59% (Fig. 8A). Consistently, the results from HPV16-infected tissues treated with L-lactate or D-lactate showed that D-lactate significantly inhibited the viral infection, while L-lactate facilitated viral infection (Fig. 8B). Taken together, our findings indicate that D-lactate is a key component for nCEVs to promote wound healing and block HPV16 infection.

## DISCUSSION

Bacteria–host cell crosstalk is a complex and intricate process (53, 54). Emerging evidence suggests that bEVs are essential mediators in cell-to-cell and cell-to-microbe communication (39). These bEVs enable the effective delivery of bioactive molecules and messages across various cell types and bacterial species within diverse ecological niches (55, 56). In this study, we demonstrated for the first time that nCEVs derived from *L. crispatus* strain are 60–80 nm size dominance of vesicles and efficiently promote cutaneous homeostasis and inhibit HPV infection *in vitro* and *in vivo*; particularly nCEVs can promote cell migration, angiogenesis, and wound healing via inducing macrophage M2-type polarization. Moreover, D-lactate is a key component responsible for the beneficial functions of nCEVs in wound healing and inhibition of HPV infection, while nIEV, derived from *L. iners* strain, contains L-lactate and exerts the opposite effect for tissue imbalance (Fig. 8C).

Vaginal epithelial cells are essential for maintaining vaginal health and form a physical barrier against pathogenic bacteria and viruses (57). Although recent studies have shown that *L. crispatus* plays a critical role in the restoration of the damaged vaginal epithelium and protection against the subsequent risk of pathogenic infections (58, 59), many other *Lactobacillus* species can produce extracellular vesicles to induce wound healing in different niches. For instance, Wu et al. demonstrated that *Lactobacillus druckerii*-derived EVs facilitate wound healing and impede scar formation in a mouse model of scleroderma (60). Wang et al. found that *Lactobacillus rhamnosus GG*-derived EVs can promote cutaneous wound healing by promoting re-epithelialization and vessel formation (61). However, it remains unclear how *L. crispatus* exerts protective functions and why different *Lactobacillus* species exert different functions. Our findings elucidate for the first time that nCEV (derived from *L. crispatus*), including D-lactate, is distinct from nIEV (derived from *L. iners*), including L-lactate, and plays a critical role in tissue homeostasis, which may serve as an innovative therapeutic strategy in regenerative medicine and cervical cancers, and merit further exploration.

Prolonged and excessive immune responses have been identified as significant risk factors compromising the vaginal barrier (62). *Lactobacillus* and other microbiota play a crucial immunomodulatory role in the human body. They help maintain the homeostasis of the immune system, uphold the integrity of the cervical epithelial barrier, and balance host defense mechanisms in the presence of microbes, all of which contribute to a healthy and functional vaginal tract (63–65). Consistent with recent research indicating that EVs derived from different *Lactobacillus* species promote the effective switch of macrophages from pro-inflammatory M1 to anti-inflammatory M2 phenotypes (66, 67), our study also showed that nCEVs could facilitate the conversion of monocytes into macrophages and promote macrophage polarization toward the M2 phenotype. Interestingly, it is worth mentioning that although nCEVs significantly promote M2 polarization, they also have minimal impact on M1 polarization. This difference could be attributed to the distinct signaling pathways involved in macrophage polarization, due to M1 polarization being typically associated with pathways such as NF-κB and STAT1, whereas M2 polarization relies more on the IL-4-/IL-13-induced STAT6 pathway (68). Thus, it is plausible that nCEVs selectively influence M2-associated pathways without significantly affecting those related to M1 polarization.

It is well known that persistent HR-HPV infection is the causative agent of cervical cancer (46), and vaginal *Lactobacillus* plays a crucial role in protecting against HPV infection, its persistence, and subsequent progression to neoplasia and cancer (69). It has been demonstrated that this protective effect could be achieved through various mechanisms, including strengthening the mucosal epithelial barrier, reducing or inhibiting chronic inflammation, and disrupting molecular pathways contributing to carcinogenesis (69, 70). Despite the various roles attributed to LEVs in recent years, how they interact with viruses remains largely underexplored and controversial. For example, although recent studies have shown that EVs from *Lactobacillus crispatus* BC5 and *Lactobacillus gasseri* BC12 can inhibit HIV infection (71) and could prevent HeLa cells from the adhesion of opportunistic pathogens (29), EVs from *Lactobacillus gasseri BC13* and *Lactobacillus crispatus BC5* did not exhibit anti-HIV properties (33). Further proteomic and metabolomic analyses revealed significant differences in the content and biological functions of EVs released by different strains of the same *Lactobacillus* species (71), indicating that the components packaged by EVs from *Lactobacillus* could be the key. In this study, we selected an *L. crispatus* strain, along with the other two *Lactobacillus* species as parallel controls, to generate nLEVs (EVs with 60–80 nm size-dominant particles) after optimized conditions, followed by investigating their roles on cutaneous wound healing and HPV infection. Supporting our hypothesis, the findings strongly showed that nCEVs significantly inhibited HPV infection *in vitro* and *in vivo*. Pretreating the target cells with nCEVs resulted in a pronounced inhibition of the virus as the treatment duration extended. This implies that nCEVs may influence the target cells by modulating intercellular junctions, the cytoskeleton, or HPV entry receptors, thereby affecting viral uptake and exerting an inhibitory effect. In contrast, pretreating HPV16 PsV with nCEVs did not significantly alter viral infectivity, indicating that nCEVs likely do not affect the structural integrity of HPV viral particles. This is distinct from EVs from *Lactobacillus crispatus *BC3 and *Lactobacillus gasseri *BC12, which can inhibit HIV infection. These EVs reduce HIV-1 infection in *ex vivo* tissues by modifying the exposure and expression of glycoprotein 120 (gp120), a key component for the virus’s attachment and entry into host cells (71). However, as HPV pseudoviruses, we used only non-replicating infected cells; it is possible that, *in vivo*, nCEVs may influence the assembly of viral particles or the release of progeny viruses in the female vagina. Additionally, since some studies have shown that certain *Lactobacillus* species can effectively reduce the risk of viral infections by modulating the immune system (72), nCEVs might help prevent HPV infection by modulating the immune activation status of the vaginal mucosa. The precise mechanisms by which nCEVs influence HPV infection—whether through a singular pathway or a combination of factors—remain unresolved and remain to be further investigated.

*Lactobacillus* produces LA in varying concentrations depending on the species and strain (52). In women with a vaginal microbiota rich in beneficial *Lactobacillus*, LA levels typically reach approximately 110 mM (1% wt/vol), which helps maintain a vaginal pH around 3.5 (73). LA exists in both L- and D-isomer forms, with different *Lactobacillus* spp. preferentially producing each isoform. At a pH below its acid dissociation constant (pKa 3.86), the protonated forms of the L- and D-isomers of LA possess direct virucidal and bactericidal properties against several sexually transmitted pathogens, including those linked to BV-associated bacteria (74), HIV (75–77). Moreover, these protonated LA isomers significantly enhance the expression of the anti-inflammatory cytokine within vaginal and cervical epithelial cells. Physiological concentrations of L- and D-LA further augment the expression of tight junction proteins (claudin and occludin), which strengthens the integrity of the cervical and vaginal epithelial cell barrier and aids in the prevention of sexually transmitted pathogen invasion (78). Despite these known benefits, the specific roles that the different isomeric forms of LA, particularly in their ionic states, play within the vaginal microenvironment are not fully understood. Additionally, it remains unclear whether different isomeric forms can exert some of the functions of nLEVs, which are known to have significant impacts on vaginal health. In our study, we found that D-lactate might be a key component for nCEVs to promote wound healing and block HPV16 infection, rather than L-lactate. We propose that D-lactate within nCEVs may facilitate wound healing and HPV16 inhibition through two interrelated yet speculative mechanisms. First, D-lactate could indirectly promote M2-like macrophage polarization, potentially via STAT6 signaling. Second, its anti-HPV16 activity might involve dual pathways: accelerating micro-wound closure to reduce viral entry points or altering host cell cytoskeletal dynamics/receptor availability to block viral uptake. These hypotheses remain untested mechanistically and require validation through targeted approaches (e.g., STAT6 inhibition or cytoskeletal disruption assays). While M2 polarization raises theoretical concerns about suppressed antiviral immunity, it is important to emphasize that our current experiments were performed exclusively in normal epithelial cells (HaCaT) under non-oncogenic conditions, and the observed HPV16 inhibition appears to be specific to early stages of infection rather than long-term viral persistence. Notably, contrasting roles of lactate isomers must be emphasized: unlike tumor-derived L-lactate (79), microbial D-lactate may contextually enhance pathogen clearance, as seen in hepatic models, where it activates Kupffer cells and restrains immunosuppressive M2 macrophages (80, 81). This duality suggests D-lactate’s effects could balance tissue repair and immune surveillance, though rigorous testing in HPV-transformed or carcinogenic microenvironments is essential to define its therapeutic scope and mitigate potential risks.

At present, while vaginal microbiota transplantation (VMT) represents an innovative approach to restoring microbial balance (82), its clinical translation faces significant challenges, including safety risks (e.g., inadvertent pathogen transmission), variable efficacy, ethical complexities in donor screening, technical demands, evolving regulatory frameworks, and high costs (83). These limitations underscore the need for safer, more scalable alternatives. LEVs, particularly those derived from beneficial vaginal bacteria such as *L. crispatus* (e.g., nCEVs in this study), emerge as a promising solution (84–86). Unlike VMT, bacterial EVs circumvent ethical and safety concerns associated with live microbial transfer while retaining their therapeutic functionality. Their inherent advantages—superior biocompatibility, low immunogenicity, and cost-effective production—position them as ideal nanotherapeutic agents (87, 88). Critically, nCEVs can be engineered to enhance targeted delivery of bioactive molecules (e.g., D-lactate), enabling precise modulation of mucosal repair and antiviral responses without the risks of whole-microbe transplantation. Our findings demonstrate that nCEVs effectively promote vaginal wound healing and inhibit HPV infection, suggesting their potential to outperform VMT in both safety and efficacy. By leveraging the therapeutic properties of bacterial EVs, this approach bypasses the logistical and ethical hurdles of VMT, offering a standardized, scalable strategy for managing vaginal dysbiosis and HPV-related pathologies.
